# Do Languages Have Exclusive Disjunctions?

**DOI:** 10.1162/opmi_a_00175

**Published:** 2024-12-15

**Authors:** Andreea C. Nicolae, Aliona Petrenco, Anastasia Tsilia, Paul Marty

**Affiliations:** Leibniz-Zentrum Allgemeine Sprachwissenschaft, Berlin, Germany; Humboldt-Universität zu Berlin, Berlin, Germany; Massachusetts Institute of Technology, Cambridge, MA, USA; Universidade de Lisboa, Centro de Linguística, Lisbon, Portugal

**Keywords:** disjunction, iterated particles, exclusivity, XOR, EXH, RSA, semantics, pragmatics

## Abstract

Most natural languages have more than one linguistic form available to express disjunction. One of these forms is often reported by native speakers to be more exclusive than the other(s) and, in recent years, it has been claimed that some languages may in fact have dedicated exclusive disjunctions. In this paper, we report on a series of experiments testing this claim across five languages of primary interest. Results show important variation in the rates of exclusive interpretation associated with the different particles used to express disjunction in these languages. Crucially, our findings show that, while complex disjunctions are usually perceived as more exclusive than their simple counterparts cross-linguistically, even the most exclusive disjunctions remain ambiguous between an inclusive and an exclusive interpretation. We discuss what factors may play a role in accounting for the gradient exclusivity effects observed in our data and how to model these effects in pragmatic and grammatical accounts of scalar implicatures.

## INTRODUCTION

Disjunctive sentences like (1) are ambiguous between an inclusive and an exclusive interpretation. Most, if not all extant accounts of this phenomenon assume that plain disjunctions like English *or* encode an inclusive meaning, yielding the literal interpretation in (1a); in positive sentences like (1), the inclusive meaning can be strengthened to an exclusive meaning via scalar inferencing, by excluding the stronger *and*-alternative to (1) (i.e., *John will visit Paris and Berlin*), yielding the enriched interpretation in (1b).

(1)  John will visit Paris or Berlin.
   a.  John will visit Paris or Berlin (possibly both).          Inclusive
   b.  John will visit Paris or Berlin, but not both.           Exclusive

English, like many other languages, has yet another way of expressing disjunction: in addition to plain *or*, we also find the morphologically complex disjunction *either_or*; similarly, in German, we find a plain disjunction, *oder*, and a more complex one, *entweder_oder*. Many languages show in fact a three-way and even four-way distinction, with two or more complex disjunctive forms available. For instance, in Russian, we find *ili*, *ili_ili*, and *libo_libo*, in Hungarian *vagy*, *vagy_vagy*, and *akár_akár*, in French *ou*, *ou_ou*, *ou bien_ou bien*, and *soit_soit*, in Romanian *sau*, *ori*, *ori_ori*, and *fie_fie*. The multiplicity of disjunctive particles in such languages raises an obvious question: do all these particles convey the same meaning?

One intuition commonly found in both the expert and non-expert literature is that the different forms available for expressing disjunction within a language relate to the extent to which they associate with an exclusive interpretation. Typically, authors of logic textbooks use, where available, the more complex disjunction(s) to exemplify the meaning of the logical exclusive operator XOR, in line with the paraphrases that linguistically naive speakers often provide for these complex forms. Similar intuitions are found in the expert literature where, for some languages, complex disjunctions have been claimed to unambiguously convey an exclusive interpretation. For instance, Spector (2014, pp. 13–18) claims that, in French, the reiterated disjunction *soit_soit*, unlike the simplex disjunction *ou*, obligatorily gives rise in non-embedded contexts to an exclusivity inference like the one in (1b). Szabolcsi (2015, pp. 194–197) extends this claim to other disjunctions with reiterated particles such as French *ou_ou*, Russian *ili_ili*, or Hungarian *vagy_vagy* and further distinguishes them from non-reiterated complex disjunctions like English *either_or*, which are assumed instead to retain both their inclusive and exclusive flavors.

Thus, according to the literature, some languages ought to have dedicated ‘exclusive’ disjunctions which obligatorily trigger the (otherwise optional) exclusivity inference associated with disjunction. Whether or not this claim is empirically correct remains, as of today, an open question which, to the best of our knowledge, has not been systematically investigated across languages using quantitative methods. The goal of this paper is to begin to close this empirical gap. In the following, we describe a simple task which can easily be deployed to test this claim cross-linguistically, and we report on a series of experiments based on this task comparing the robustness of the exclusive interpretation associated with simplex vs. complex disjunctions (one vs. two overt particles) in five different languages, four of which express complex disjunctions via fully-iterated particle constructions.

## EXPERIMENTS

We investigated the robustness of the exclusive interpretation associated with different disjunction markers within and across five languages: English, French, Romanian, Russian, and Greek. For each of these languages, we chose three of the most commonly used disjunctive markers, one simplex and two complex, with the sole exception of English which only employs one complex disjunction, namely *either_or*. The disjunctive constructions tested in all five languages are shown in Table 1. Each language group was tested in a separate experiment, thus yielding a total of 5 experiments.

**Table 1.** Schematic description of the disjunctive constructions tested in all five languages under investigation; D1 are simplex disjunctions whereas D2 and D3 are all complex disjunctions.

	D1	D2	D3
English	**A** *or* **B**	*either* **A** *or* **B**	n/a
French	**A** *ou* **B**	*ou bien* **A** *ou bien* **B**	*soit* **A** *soit* **B**
Romanian	**A** *sau* **B**	*fie* **A** *fie* **B**	*ori* **A** *ori* **B**
Russian	**A** *ili* **B**	*libo* **A** *libo* **B**	*ili* **A** *ili* **B**
Greek	**A** *i* **B**	*i* **A** *i* **B**	*ite* **A** *ite* **B**

**Figure 1.**
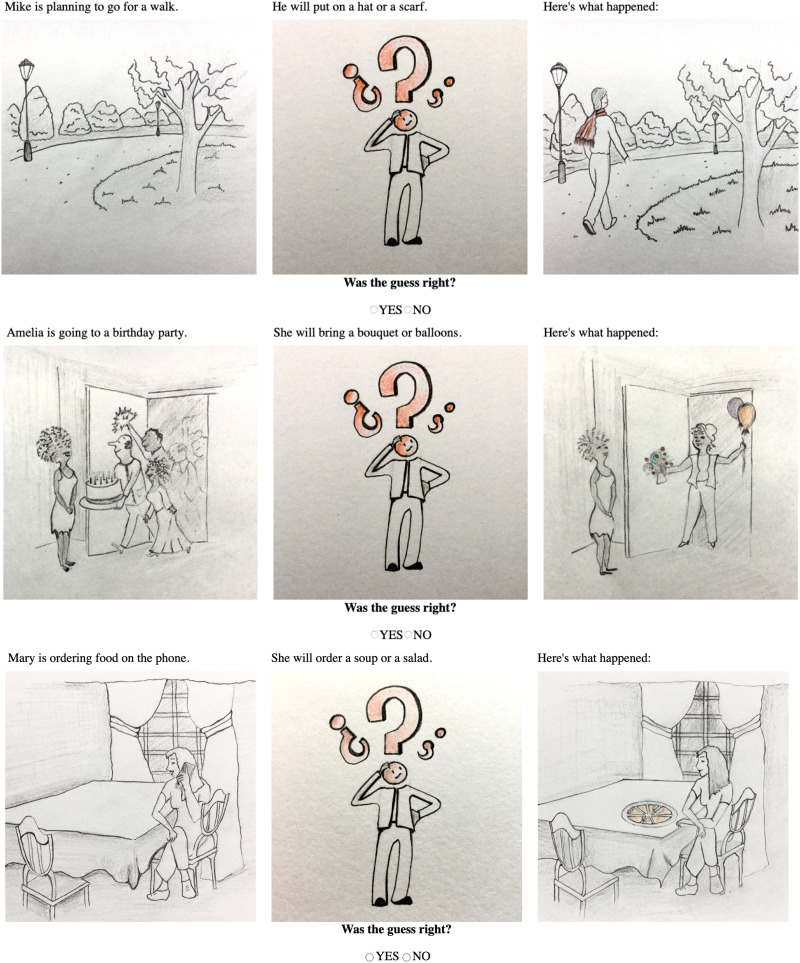
Example scenarios used in the true (at the top), target (in the middle) and false (at the bottom) conditions. Scenarios were unfolded before the participants, one scene at a time.

All experiments used the same task and were based on the same materials and design. For our purposes, we adapted the truth-value judgment paradigm (Crain & Thornton, 2000) into a Yes-No task featuring the so-called ‘prediction’ mode (Tieu et al., 2017, 2019, a.o.). Participants were presented with unfolding scenarios like those in Figure 1 and had to decide whether the character’s guess made in the second scene was right or wrong, given the outcome represented in the third and final scene. Scenarios were unfolded before the participants, one scene at a time, with the disjunctive test sentences being uttered in the second scene, before the last scene was revealed on the screen.

The rationale for implementing the task in prediction mode was to obtain more pristine measures of the exclusive interpretations of interest by preventing other pragmatic inferences associated with disjunction from affecting participants’ judgments. In particular, it is well-known that, in addition to and independent of exclusivity inferences, disjunctive sentences of the form ‘*p* or *q*’ readily give rise to speaker-oriented ignorance inferences about ‘*p*’ and about ‘*q*’ (Gazdar, 1979; Marty & Romoli, 2022a, 2022b; Meyer, 2013; Sauerland, 2004; Simons, 2001; Singh, 2010, a.o.).^1^ These inferences make an utterance of ‘*p* or *q*’ infelicitous in contexts where the speaker can reasonably be assumed to know which of the disjuncts holds. In our case, for instance, such contexts would likely be obtained if the character were to utter ‘*p* or *q*’ after, rather than before witnessing the relevant event unfold—that is, if the character’s statements were first-hand descriptions, rather than mere predictions of the outcome. Note that, in this alternative setup, participants’ rejection of an utterance of ‘*p* or *q*’ when both *p* and *q* are true would then have two possible sources: it could result from an exclusive interpretation of the disjunction or from conflicting ignorance inferences. The prediction mode allowed us to circumvent this methodological issue by making it constitutive of the task that the character was always ignorant of the outcome. As a result, participants’ judgments in our experiments should only reflect their intuitions as to whether the character’s assertion is right or wrong given the outcome.

### Participants

564 participants were recruited online using the recruiting platform Prolific (minimum prior approval rate: 90%; nationality, country of birth, and first language were also used as prescreening criteria; the values for these criteria were set based on the language being tested). The sample of subjects included native speakers of English (*n* = 90), French (*n* = 127), Romanian (*n* = 111), Russian (*n* = 107), and Greek (*n* = 129), yielding between 30 to 45 subjects per disjunction in each language group. Participants were paid approximately £1.7 for their participation and average completion time was about 9 minutes (£8/hr).

### Materials and Design

Each trial consisted of a scenario unfolding over three scenes, where the sentences to be judged by the participants appeared in the second scene before the third and last scene was displayed on the screen (see examples in Figure 1). The structure of the scenarios was the same across all trials: the first scene set the stage of a story by displaying a picture together with a short sentence describing a future event; the second scene showed a character making a guess about what was going to happen next in that story in relation to the relevant event; finally, the third and last scene revealed the outcome of the story by means of a novel picture accompanied by the lead-up ‘Here’s what happened’.

Target sentences were disjunctive sentences of the form *[Pronoun] will [verb] [(D) A D B]* such as *She will bring (either) a bouquet or balloons*. The [pronoun] term always agreed with the subject of the sentence displayed in the first scene; the [verb] term was an action verb; the disjunctive phrase [(D) A D B] involved a simplex or complex disjunction type connecting two common nouns (A and B) denoting inanimate, concrete objects that are semantically related (e.g., *apple tree* and *cherry tree*, *scarf* and *socks*). The disjunction marker used in these sentences was manipulated between subjects so as not to encourage implicit, comparative judgments between disjunctive constructions.^2^ Thus, participants in our experiments only ever saw target sentences with one and the same disjunctive marker.

Target sentences were presented with one of three possible story outcomes obtained by manipulating the visual content of the final scene picture. In the target conditions, the two objects mentioned in the sentence appeared on the final image, making the sentence false on an exclusive interpretation (expected answer: ‘No’), but true on an inclusive interpretation (expected answer: ‘Yes’). In the false and true control conditions, neither or only one of the two objects mentioned in the sentence appeared on the final image, making the sentence unambiguously false or true (correct answer: ‘No’ and ‘Yes’, respectively). There were 3 test items per condition, hence 9 test items in total. The linguistic materials in the test items were created by varying content and function words that were not critical to our manipulation. The linguistic stimuli were designed so as to make the test items relatively uniform across the board and minimize the risk that contextual factors bias participants towards one particular interpretation of the disjunction.^3^ An illustration of the linguistic materials used in the test items is given in Table 2.

**Table 2.** Example linguistic stimuli used in the test items. This list corresponds to the test items for English *or*. The first sentence corresponds to the short description accompanying the first picture and the second to the character’s assertion. This list was adapted in each survey to the language and disjunction type of interest. In the survey, the order of the items was randomized.

Item	Linguistic stimuli (initial sentence ⊳ character’s assertion)	Condition
T1	Laura decided to start a garden. ⊳ She will plant a cherry or an apple tree.	Target
T2	Jen wants to decorate the wall. ⊳ She will hang a poster or a clock.	True
T3	John is going to the market. ⊳ He will buy a watermelon or a pumpkin.	False
T4	Amelia is going to a birthday party. ⊳ She will bring a bouquet or balloons.	Target
T5	Mike is planning to go for a walk. ⊳ He will put on a hat or a scarf.	True
T6	Oscar is going to a party. ⊳ He will bring a cake or chips.	False
T7	Rachel is invited to a gala. ⊳ She will wear a necklace or a hat.	Target
T8	Gabe wants to relax this evening. ⊳ He will knit a scarf or socks.	True
T9	Mary is ordering food on the phone. ⊳ She will order a soup or a salad.	False

Each survey further included 18 non-disjunctive filler items (6 true, 6 false and 6 open to interpretation), thus yielding a total of 27 items. These items involved non-disjunctive sentences unrelated to our experimental purposes and were added to make the target items less visible across the experiment. All linguistic materials were originally created in English and then adapted and translated into French, Romanian, Russian, and Greek by linguistically-trained native speakers.

### Procedure

The experiment was run on PCIbex Farm (Zehr & Schwarz, 2018). At the beginning of the survey, participants were presented with the following cover story (English version; all materials were translated by native speakers into the corresponding languages):

*Kate and Henry are two friends who like playing games. You will now witness one of their games. The rules are as follows: Kate draws two pictures and doesn’t show them to Henry. The first picture depicts a situation and comes with a sentence describing it. The second picture depicts a follow-up scene. She shows Henry the first picture and asks him to make a guess about what’s going to happen. Then, Kate presents the second picture with the follow-up scene.*

Participants were tasked to judge whether Henry’s guess was right given the outcome revealed in the follow-up scene; they were instructed to click on ‘Yes’ if they considered the guess right and, otherwise, to click on ‘No’. Following these instructions, participants were asked two demographic questions before continuing to the experiment.

For each participant in each language group, it was pseudo-randomly determined which disjunction type would appear in the target sentences that they would see. Participants started the experiment with two practice trials and then completed 27 items presented to them in a random order. In every trial, participants had to click on a ‘Next’ button to advance from one scene onto the next. Previous scenes remained on the screen throughout the unfolding scenario. After the last scene was displayed, the question ‘Was the guess right?’ appeared underneath. Participants provided their answer by clicking with the mouse on one of two response buttons labeled ‘Yes’ and ‘No’, respectively. Items remained on the screen until participants provided their response. At the end of the survey, participants were asked to fill out a short feedback form.

### Software

Data processing, analysis and visualization were carried out in the R statistical environment (R Core Team, 2023) using the tidyverse (Wickham et al., 2019), Hmisc (Harrell, 2023), dplyr (Wickham et al., 2023), lme4 (Bates et al., 2015), car (Fox & Weisberg, 2019), DHARMa (Hartig, 2022), ggplot2 (Wickham, 2016), janitor (Firke, 2023), ggpattern (FC et al., 2022) and RColor-Brewer (Neuwirth, 2022) packages for the R statistics program.

### Data Preparation

Responses from 37 subjects (6.5% of the sample) were removed prior to analyses because their performance on the true and false control trials did not reach the pre-established threshold of 80% accuracy (i.e., at least 5 out of 6 trials correct).

### Data Analyses

#### Main Analysis.

**Figure 2.**
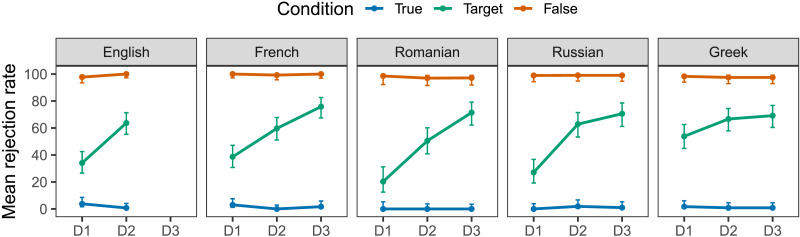
Mean rejection rate (i.e., percentage of ‘No’ responses) to the test items by language, condition and disjunction type. In the target conditions, this measure stands proxy for the rate of exclusive interpretation. Error bars represent 95% binomial confidence interval estimates.

Responses to the test items are summarized in Figure 2. As expected, the mean rejection rates (i.e., percentages of ‘No’ responses) were lowest in the true conditions (all *M*s > 4%) and highest in the false conditions (all *M*s > 96%). In the target conditions, all disjunctions in all five languages received an intermediate rejection rate, in-between those observed for their true and false baselines. The mean rejection rates for D2 and D3 were relatively uniform across languages, with 8 out of 9 instances in the 60–75% range; the rates for D1 showed more variations, ranging from 20% in Romanian to 54% in Greek. These response patterns are expected only if the disjunctions of interest are assumed to be ambiguous between an inclusive and an exclusive reading.

For each language, we fitted a GLMER model (logit link function), predicting responses in the target conditions from the fixed effect of disjunction (3 levels: D1, D2 and D3; dummy coded). Each model included by-participant and by-item random variance for the intercept, which was the maximal random effect structure supported by the data.^4^ Each model was compared to a null model missing only the fixed effect of disjunction. The model with the fixed effect of disjunction was found to provide a significantly better fit to the data compared to the null model for English ($\chi_{1}^{2}$ = 25.26, *p* < .001), French ($\chi_{2}^{2}$ = 21.06, *p* < .001), Romanian ($\chi_{2}^{2}$ = 34.63, *p* < .001), Russian ($\chi_{2}^{2}$ = 36.51, *p* < .001) but not for Greek ($\chi_{2}^{2}$ = 4.64, *p* = 0.09), where the mean rejection rate for D1 was only marginally lower than those for D2 and D3. In all other languages with reiterated disjunctions (French, Romanian, and Russian), both D2 and D3 yielded significantly higher rejection rates than D1 (all *β*s > 3.27, all *p*s < .05). Further reliable contrasts were found between D2 and D3 in French (*β* = 2.55, *p* =.05) and Romanian (*β* = 7.23, *p* < .05), showing that distinct reiterated disjunctions in these languages prompted exclusive interpretations to a different extent. No such contrast between D2 and D3 was found in Russian (*β* = 0.59, *p* = 0.7).

#### By-Item and By-Participant Variability.

**Figure 3.**
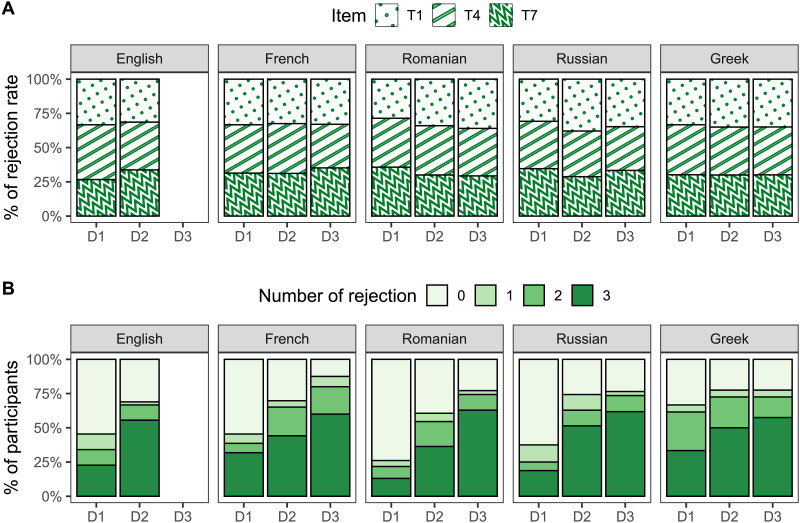
**(A)** Relative frequency of ‘No’ responses as a function of the target item, language, and disjunction type. **(B)** Relative frequency of participants as a function of the number of rejections in the target conditions, language, and disjunction type.

Figure 3A shows the relative frequency of ‘No’ responses as a function of the target item (T1, T4 and T7; see Table 2), language and disjunction type. In the whole data set, T1 contributed 34% of the ‘No’ responses in the target conditions, T4 35% and T7 31%. The maximal difference between these items per language across all disjunctions ranged from 1% (French) to 6% (English). A visual inspection of the stacked barplot in Figure 3A suggests that these observations hold across the board: for each disjunction type in all five languages, T1, T4, and T7 were rejected to a very similar extent, meaning that each of them contributed roughly equally to the mean rejection rates reported above in Figure 2. We take these results to show that variation in the non-critical materials used in the target items, i.e., beyond the manipulation of the disjunctive marker, had no remarkable effect on participants’ judgments of exclusivity.

Figure 3B shows the relative frequency of participants as a function of the number of rejections in the target conditions (0, 1, 2, or 3), language and disjunction type.^5^ Overall, about 80% of the participants always accepted (inclusive responders) or always rejected (exclusive responders) the target items. This behavior was predominant in every language group, with a prevalence ranging from 73% (Greek) to 82% (English and Romanian). In line with the results from the main analysis, the proportion of inclusive responders was overall higher for D1 (54%) than for both D2 (30%) and D3 (20%) whereas the proportion of exclusive responders was overall higher for both D3 (63%) and D2 (48%) than for D1 (25%). Taken together, these results show that the vast majority of the participants consistently responded in accordance with their initial interpretive preference, where this initial preference varied as a function of the disjunctive marker involved.

## DISCUSSION

The findings are threefold. First, all the disjunctions tested in this study yielded ambiguity patterns showing that, no matter how ‘exclusive’ they feel, they all retain an inclusive interpretation to some extent. Second, complex disjunctions generally yielded higher rates of exclusive interpretations than simplex ones across languages. Third, the results show that speakers’ propensity to interpret a disjunction exclusively varies substantially: (i) there is wide cross-linguistic variation in how exclusive simplex disjunctions are interpreted (e.g., Romanian vs. Greek), and (ii) further contrasts may exist among complex disjunctions within the same language (e.g., in French and Romanian). Consistent with the layman’s intuition, these findings support the idea that complex disjunctions are more strongly associated with an exclusive interpretation than simplex ones. However, they disconfirm the stronger claim that reiterated disjunctions in languages like French, Russian, or Romanian are dedicated ‘exclusive’ disjunctions categorically distinct from simplex ones. It is possible, of course, that such disjunctions exist in some language(s) not included in this study. In particular, the sample of languages investigated here, while representative of those which have been discussed in the expert literature and for which the claim that we tested was made explicitly, remains limited to Indo-European languages. Outside the Indo-European family, we also find languages such as Kannada that use simple and fully-iterated particles to express disjunction, with some of them being claimed to be ambiguous and others exclusive, e.g., *-oo_-oo* vs. *illa_illa* in Kannada (Amritavalli, 2003; Szabolcsi, 2015). The materials and task that we introduced in this article can be used to expand the present investigation to these languages and address these limitations.

Let us now turn to the consequences of these findings for current approaches to reiterated disjunctions, focusing on Spector (2014)’s analysis of French *soit_soit*, which Szabolcsi (2015) proposes to extend to other fully-iterated particle constructions like Russian *ili_ili*, among others. Spector (2014)’s analysis of French *soit_soit* relies on two main assumptions. First, it assumes *soit_soit* must associate with an occurrence of the exhaustivity operator exh, a silent grammatical operator akin in meaning to *only* (Chierchia et al., 2012). Specifically, it assumes that the only licit parse for an utterance of ‘soit *A* soit *B*’ is one where *soit_soit* occurs in the scope of exh, that is, exh[soit *A* soit *B*]. Second, it assumes that the distribution of the exhaustivity operator is regulated by an Economy Constraint which states that an occurrence of exh in a sentence *S* is unlicensed if eliminating this occurrence would lead to a sentence *S*′ such that *S*′ entails *S* (Chierchia et al., 2012; Fox & Spector, 2018); in effect, this constraint rules out any parse exh[*S*′] that would result in a meaning equivalent to or weaker than the meaning of *S*′ alone. In the case of exh[soit *A* soit *B*], this constraint is met by excluding the conjunctive alternative to exh’s prejacent, i.e., by deriving the exclusive reading for disjunction.

As it is formulated, Spector’s analysis predicts disjunctive markers like *soit_soit* to always give rise to some form of meaning strengthening. In non-embedded contexts, it boils down to saying that such disjunctions should be interpreted exclusively. As should be clear from the above, this prediction, in its categorical form, is not borne out by the results of our experiments. We note, however, that Spector’s account can be minimally amended to capture our data. Specifically, the association between reiterated disjunctions and exh can be reformulated as an interpretative preference rather than a categorical requirement, e.g., *soit_soit* occurs preferentially (rather than obligatorily) in the scope of an exhaustivity operator. We discuss possible reasons for this interpretative preference below. For now, we observe that this amendment would lead to novel predictions departing slightly from Spector’s claims. In particular, Spector argues that *soit_soit* cannot occur in global downward-entailing (DE) contexts. This observation is illustrated in (2) (Spector, 2014, ex. 7a) and (9a)): unlike *ou* in (2a), *soit_soit* is reported to be unacceptable in (2b), where it is embedded under negation. This restriction on the distribution of *soit_soit* is immediately accounted for on Spector’s analysis, i.e., if we assume that *soit_soit* must occur in the scope of exh.

(2)   a. Je ne pense pas que Jacques ait invité Anne **ou** Paul à dner.
       Can mean: ‘I don’t think that Jacques invited either for dinner.’
     b. *Je ne pense pas que Jacques ait invité **soit** Anne **soit** Paul à dner.
       Cannot mean: ‘I don’t think that Jacques invited either for dinner.’

Crucially, if we loosen Spector’s requirement and assume that reiterated disjunctions merely favor exclusive readings, as our data suggest, we would now expect to find some variability in speakers’ judgements regarding the (un-)acceptability of *soit_soit* in DE-contexts, just like we found some variability in speakers’ judgements of exclusivity. Specifically, we would expect the two types of judgments to be correlated: the less speakers perceive *soit_soit*-type disjunctions as exclusive in non-embedded contexts, the more they should accept them in global DE-contexts. Whether this prediction is borne out is beyond the scope of this paper but we believe that testing the presence and strength of this correlation would help clarify whether the propensity of certain disjunctions to give rise to exclusivity inferences and their behaving like positive polarity items should be conceived as two sides of the same coin, as per Spector’s proposal.^6^

At a more general level, our findings show that exclusivity inferences are not a categorical phenomenon and raise the question of what factors may play a role in accounting for the gradient exclusivity effects (or lack thereof) that we observed in our data and how to model these effects in a formal framework. That is, how do we account for the fact that, everything else being equal, listeners are more or less likely to derive these implicatures depending on the disjunctive construction involved? One way to tackle this question, as we shall argue, is to actually look into the possible factors driving speakers’ likely production choices in the first place, i.e., their reasoning for favoring one type of disjunction over another, and thus start by tackling another pressing question: given the general pragmatic preference for less costly (e.g., shorter) utterances, why would speakers ever choose to produce a (more) complex disjunction, if complex and simplex disjunctions have the same literal meaning and license the same form(s) of scalar enrichment?

We would like to suggest here that the production of complex disjunctions involves in fact a trade-off between their higher morpho-syntactic cost and their greater impact on certain aspects of discourse affecting utterance interpretation. Specifically, we propose that, compared to simplex disjunctions, complex disjunctions facilitate the realization of specific prosodic patterns, which speakers may use to signal the Question Under Discussion (Roberts, 2012) they intend to address more effectively and, in particular, to bring out QUDs that make the stronger alternatives to the disjunction more relevant. To illustrate this point, let us consider the typical phonological realization of a declarative sentence with *either_or* in English. Intuitions suggest that, compared to *or*, *either_or* facilitates, if not favors, narrow focus on each disjunct, possibly setting up the disjuncts in a contrastive focus configuration, as illustrated in (3) (where capitalization indicates that the word is accented).^7^

(3)  **Narrow/Contrastive focus**
   a.  ?John will visit Paris or Berlin.
       (cf. broad focus: John will visit Paris or Berlin)
   b.  John will visit either Paris or Berlin.

We take the focus structure associated with *either_or*-sentences like (3b), where both disjuncts receive prosodic prominence, to signal that the (implicit) QUD is more likely one that makes the conjunctive *and*-alternative relevant (e.g., *Will John visit Paris and Berlin?*) rather than one that does not (e.g., *Will John visit any capital city?*).^8^ Importantly, by increasing the relevance of the *and*-alternative, this type of QUD also increases the likelihood that the exclusivity inference is derived. Intuitively, the reason is that, under a QUD like *Will John visit Paris and Berlin?*, knowing whether the *and*-alternative is true or false would resolve the QUD, making it therefore higly relevant. By contrast, under a QUD like *Will John visit any capital city?*, this same alternative becomes somewhat less relevant as it would resolve the QUD only if it is true (see Degen, 2023; Kursat & Degen, 2020; Marty et al., 2024 for discussion of related examples involving *some* and numerals). Assuming that the speaker is trying to be as informative as possible with respect to the partition of the context set induced by the QUD, the former type of QUD is thus more likely to elicit an exclusive inference than the latter.

On this proposal, speakers’ choice to use a more complex disjunction should thus be conceived as a strategic choice to optimize communicative effectiveness: the richer structure of complex disjunctions, while resulting in higher production costs, also allows the realization of more distinctive prosodic patterns which, we argue, favor QUDs that promote the relevance of the conjunctive alternative to the disjunction. On the interpretation side, the perceived increase in the alternative’s relevance may, in turn, increase the speaker’s certainty that the context they are in is one that supports the implicatures associated with the alternative of interest, leading to the conclusion that an exclusive reading of the disjunction is most likely intended by the speaker. A summary of the key differences between simplex and complex disjunctions discussed above is given in Table 3.

**Table 3.** A comparison between simplex and complex disjunctions illustrating the key factors in the trade-off between production cost and discourse impact.

	Simplex disjunction (*or*)	Complex disjunction (*either_or*)
Utterance cost	Less costly	More costly
Impact on discourse (QUD)	Lower	Higher
Relevance of alternatives	Lower	Higher
Scalar implicatures	Less likely	More likely

This proposal effectively accounts for the finding that, across the languages we tested, complex disjunctions generally yielded higher rates of exclusive interpretations than their simpler counterparts. However, it does not capture all the variations that we observed across disjunctions and languages in our data. Specifically, it does not explain why, within a given language, two complex disjunctions may differ in exclusivity, as observed in French and Romanian. Nor does it explain why simplex and complex disjunctions need not differ much in this regard, as observed in Greek. We note, however, that the present proposal could in principle be adjusted to overcome these limitations and accommodate such cross-linguistic variations, whether by integrating other factors that may affect the ability of disjunctions to bring alternatives into focus or by refining the metrics used to evaluate their effects.^9^ For instance, within a given language, two disjunctions of comparable complexity may still show subtle differences in their typical prosodic realization, e.g., in the degree of emphasis they allow; similarly, other prosodic properties of the language may make it so that simple disjunctive constructions need not differ much from their complex variants in their ability to raise alternative relevance through prosodic marking.^10^ While these remarks remain speculative for now, we believe that investigating further the relationship between the prosodic realization and interpretation of disjunctive constructions is a promising next step to better understand the source of the gradient exclusivity effects that we found.

From a theoretical perspective, our core proposal can be integrated with both pragmatic and grammatical accounts to scalar implicatures. In the pragmatic tradition, probabilistic accounts like the Rational Speech Act (RSA) framework (Frank & Goodman, 2012; Goodman & Frank, 2016; Scontras et al., 2021) provide a particularly suitable approach to formalize and implement the factors that we have identified (see Bergen et al., 2016; Goodman & Stuhlmüller, 2013; Rohde et al., 2012 for applications of the RSA framework to implicatures and Degen (2023) for an overview of these applications).^11^ In a nutshell, in standard RSA models, the pragmatic speaker reasons about the interpretation choices of a listener interpreting utterances according to their literal semantics and chooses utterances that balance cost and informativeness. The pragmatic listener, in turn, interprets utterances by considering how likely the pragmatic speaker would have been to choose the observed utterance given different possible meanings they might want to communicate. In our case, this design immediately captures our intuition that, when selecting disjunctions, (pragmatic) speakers consider their varying complexity in light of their effect on interpretation: where choosing a simpler disjunction would reduce the utterance cost, a more complex one may be preferred if it is more efficient at communicating the speaker’s intended meaning. Standard RSA has also been extended to account for other forms of context dependence, like the sensitivity to the QUD (Kao & Goodman, 2015; Kao et al., 2014; see also Scontras et al., 2021 and Degen, 2023 for an overview). Current implementations of this idea model the QUD in terms of its effect on the assumed meaning space, where an increase in alternative relevance translates into an increase in informativeness relative to the cells of the partition induced by the QUD. Future work could thus capitalize on such proposals to formalize the second key ingredient of our proposal and explain how different disjunction constructions may differ in informativeness despite having the same literal semantics.

In the grammatical tradition, the factors that we have identified would most naturally fit into a theory of ambiguity resolution modeling the effects of different linguistic and contextual cues on listeners’ parsing strategies and, specifically, on their decision to insert or not insert exh.^12^ Previous work in this tradition has emphasized for instance the focus-sensitive nature of exh and the role of focus marking in licensing its insertion (Bade, 2016; Bade & Sachs, 2019; Fox & Spector, 2018) as well as determining the set of alternatives to its prejacent (Fox & Katzir, 2011). Building on this work, one could hypothesize that, in disjunctive constructions, the placement of focus on each disjunct may act as a perceptual cue signaling the presence and scope of the (otherwise silent) exh-operator and bias listeners toward a parse where each disjunct is locally exhaustified. To illustrate, consider again the example in (3b), where both disjuncts are focus-marked. On the grammatical view, this kind of prosodic structure could be taken to favor a parse of (3b) where an occurrence of exh is embedded in each disjunct, (4a). On this parse, the set of formal alternatives would then correspond to the independent disjuncts themselves, (4b). As it is easy to verify, the result of exhaustifying the meaning of each disjunct, using the other disjunct as an alternative, delivers a global meaning that entails the exclusivity inference we were after, (4c).

(4)   a. [exh John will visit either Paris] or [exh 〈he will visit〉 Berlin].
     b. Alternatives = {John will visit Paris, John will visit Berlin}
     c. ⇝ John will visit Paris but not Berlin, or he will visit Berlin but not Paris

For now, our data leaves open the question of whether complex disjunctions favor strengthening in general or local strengthening in particular. This empirical question could be addressed in future work by investigating the interpretation of simplex and complex disjunctions in other, embedded environments where global and local strengthening results in logically distinct exclusivity inferences and thus can be teased apart, e.g., when disjunction is embedded under a universal quantifier (e.g., *Every student will visit (either) Paris or Berlin*).

Finally, let us circle back to the question we started with: do all disjunction particles convey the same meaning? For the time being, our results show that all disjunctions in all five languages we tested are ambiguous between an inclusive and an exclusive interpretation and that they may, but need not differ in terms of how exclusive they are. This leaves us with an open question regarding the multiplicity of disjunction particles in languages like Russian and Greek where, in contrast to languages like French and Romanian, we do not see gradient effects of exclusivity all the way: why would a language have three or more ways of expressing disjunction if only one or two gradients of exclusivity tend to be expressed? We believe that future work may shed light on this question by considering other inference types which may arise from disjunctive sentences. One prime example is the ignorance inferences which we mentioned before, on which the speaker is taken to be ignorant as to which of the disjuncts holds. Another example is the ‘exhaustivity’ implicatures, on which contextually relevant *ad hoc* alternatives not mentioned in the disjuncts (e.g., Madrid, Lisbon) are taken to be false. For the sentence in (1), these inferences can be paraphrased as shown in (5a) and (5b), respectively.

(5)   a. The speaker doesn’t know which of Paris or Berlin John will visit.   Ignorance
     b. John will not visit any other city besides Paris or Berlin.      Exhaustivity

Note that the inferences above do not carry any particular commitment as to whether or not exclusivity holds: they are independently and mutually compatible with John visiting only one or both cities. In principle then, two disjunctions similar in terms of how exclusive they are, may still differ in their ability to prompt these other inferences. Whether or not this is the case is an empirical question which we shall leave to future work.

## CONCLUDING REMARKS

Variability in rates of scalar implicatures has been observed between individuals, participant groups, experimental tasks, linguistic environments and scalar expressions. Our results add to this growing body of knowledge on scalar variability by showing that, all else being equal, rates of scalar implicatures may also widely vary across different linguistic realizations of the same underlying concept, in the case at hand, disjunction. Previous experimental work in a similar vein comes from the domain of existential quantifiers which, just like disjunctions, can have multiple realizations within the same language (e.g., *quelques* and *certains* in French; *sommige* and *enkele* in Dutch) and have been found to differ in their propensity to give rise to scalar implicatures (see Pouscoulous et al., 2007 for French and Banga et al., 2009 for Dutch). The present study offers the first large-scale investigation of this phenomenon where the same task and design were employed to test differences both within and across languages.

## ACKNOWLEDGMENTS

The authors are grateful to the audience at Sinn und Bedeutung in Bochum, the semantics colloquium in Göttingen, the workshop on Logic, Grammar and Meaning in Milan, the Brown Bag lunch series in Berlin and the Nihil workshop in Amsterdam, where different incarnations of the present study have been presented. We are also thankful to Camelia Bleotu for helping us with the Romanian translations and to Monica Casa for her invaluable help with corpus data, which unfortunately did not make it into the final version of the paper. Special thanks go to Maria Aloni, Nina Haslinger, Clemens Mayr, Uli Sauerland, Viola Schmitt, Benjamin Spector and Yasu Sudo for useful discussion of the theoretical implications of our findings.

## FUNDING INFORMATION

This project has received funding from the European Research Council (ERC) under the European Union’s Horizon 2020 research and innovation programme (grant agreement No. 856421). Further support was provided by the DFG grant NI-1850/2-1, awarded to Andreea C. Nicolae, and by Portuguese national funds through Fundação para a Ciência e Tecnologia, under project UIDB/00214/2020, of Center of Linguistics of the University of Lisbon.

## AUTHOR CONTRIBUTIONS

A.C.N.: Conceptualization; Formal analysis; Investigation; Methodology; Resources; Supervision; Validation; Writing – original draft; Writing – review & editing. A.P.: Investigation; Methodology; Resources. A.T.: Methodology; Resources. P.M.: Formal analysis; Methodology; Validation; Visualization; Writing – original draft; Writing – review & editing.

## DATA AVAILABILITY STATEMENT

Stimuli, data files and analysis output associated with the experiments reported in this paper are available open access on the Open Science Framework platform at https://osf.io/7e8w4/. The research hypotheses and analytical plan for the initial experiment with English speakers was pre-registered before data collection, in December 2020, using the AsPredicted.org template via the Open Science Framework at https://osf.io/z26ct.

## ETHICS AND CONSENT

The study was approved by the UCL Research Ethics Committee (UCL REC) under approval protocol for non invasive research on healthy adults LING-2021-01-21. The experiments were conducted in accordance with the Declaration of Helsinki. Written informed consent was obtained from all participants prior to experimentation, based on a detailed description of the experimental procedure, the reward scheme, and our use of the submitted data. Wherever relevant, the approved English version of the informed consent document was translated into the participant’s native language. Data were collected and stored in accordance with the provisions of Data Protection Act 2018.

## Notes

^1^ In the literature, these inferences are generally treated as scalar implicatures arising from the comparison between the whole disjunction, ‘*p* or *q*’, and the independent disjuncts, namely *p* and *q*. The question of how exactly these inferences are derived is immaterial to the present discussion. What is important to us is that the presence of these inferences makes the use of the more typical description mode ill-suited for our purposes since it would not allow a one-to-one mapping between response type (Yes vs. No) and disjunction interpretation (inclusive vs. exclusive) in the critical conditions where both disjuncts are true.^2^ Nicolae and Sauerland (2016) investigated the acceptability of the exclusivity inference associated with *or* and *either_or* using the inferential task paradigm. They found that participants were less likely to endorse the exclusivity inference for *or* when disjunction type was manipulated within-subject, compared to when it was manipulated between-subject. These results suggest that speakers’ judgments for one disjunctive marker may be affected by the presentation of other disjunctive markers within the same experiment.^3^ As an anonymous reviewer pointed out to us, the interpretation of disjunction may be affected by factors such as the taxonomy relationship between the two objects connected by the disjunction. Intuitively, the disjunction in the sentence *He will wear a neckerchief or a scarf* is more likely to receive an exclusive interpretation than in the sentence *He will wear a necklace or a scarf*, simply because it is less likely to wear a neckerchief and a scarf at the same time. In designing the items, we tried as much as possible to avoid situations where world knowledge alone would lead to the expectation that the two relevant events are inclusive/exclusive.^4^ The model for French triggered a singular fit warning due to the by-item random variance for the intercept being estimated very near zero. As a sanity check, this model was refitted without the random intercept for items. The values of the coefficients of the refitted model were the same as before. While this warning only arose for this model, we note that the estimated variance for the item random effect was relatively small in all models. These observations are consistent with the finding we report on below that, independent of the language and disjunction type, there was generally very little variation in responses across test items.^5^ There were 3 test items per condition; thus, the number of ‘No’ responses in the target conditions for any participant is between 0 and 3, where 0 means that the participant always responded consistent with an inclusive interpretation and 3 that they always responded consistent with an exclusive interpretation.^6^ Nicolae (2017) shows that the positive polarity behavior of certain disjunctions could also be explained in reference to the ignorance inferences associated with disjunction, if we assume that these inferences are derived in the grammar as well; but see also Nicolae (2012) for an alternative account of positive polarity that treats it as independent from both exclusivity and ignorance inferences.^7^ While this contrast may be subtle in post-verbal position, it becomes more pronounced in pre-verbal position, e.g., ^??^John *or* Sue *will visit Paris* vs. *Either* John *or* Sue *will visit Paris*. In addition, we note that, with simplex disjunctions, it is also possible to focus the disjunctive marker itself, as in *John* or *Sue will visit Paris*. This prosodic realization, however, remains marked in English and only seems fully felicitous as a correction to a previous statement.^8^ We remain agnostic as to whether the focus structure illustrated in (3b) directly raises the relevance of the conjunctive alternative, or does so indirectly, by raising the relevance of the independent disjuncts. We note, however, that this distinction becomes moot if we assume that relevance is closed under conjunction (i.e., if *p*, *q* are relevant, so is *p* ∧ *q*), as proposed in Fox (2007) and Fox and Katzir (2011) (attributing the proposal to class notes from a 1997 seminar taught by Kai von Fintel and Irene Heim at MIT).^9^ This is also true of the metrics we used for evaluating disjunction cost, on which the cost of a disjunction reduces to its morpho-syntactic complexity and prosodic correlates. As an anonymous reviewer pointed out to us however, in French, *ou bien_ou bien* (D2) appears to be more costly than *soit_soit* (D3), at least in term of words/syllables. For now, it is an open question whether other parameters beyond those that we identified may play a significant role in the calculation of disjunction cost.^10^ Another possible source of cross-linguistic variation could be the interaction between prosodic realization and reading, especially on a computer screen. That is, it could be the case that the extent to which prosody is realized, internally, during reading substantially varies not only between individuals but also between language groups. We thank an anonymous reviewer for sharing this insight.^11^ We cannot do justice in this paper to the sophisticated machinery deployed in standard RSA and its many extensions. Our goal here is modestly to point to components of RSA models where the factors that we have identified would most naturally fit. We refer the reader to the references therein for detailed discussion.^12^ Some grammatical accounts assume instead that the insertion of exh is syntactically mandatory (Magri, 2009, 2011; Marty, 2017; Marty & Romoli, 2021). On these accounts, the decision to derive or not derive an implicature usually boils down to assessing whether or not the alternative it is based on is relevant given the utterance context. As far as we can see, our proposal is also compatible with these accounts, to the extent that the notion of relevance is treated as gradient rather than categorical.
